# Exploring sociodemographic and nutrition-related correlates of meal-kit use across five countries: findings from the International Food Policy Study

**DOI:** 10.1017/S1368980025101584

**Published:** 2025-12-23

**Authors:** Liza Boyar, Christine M. White, Lana Vanderlee, Jean Adams, Martin White, Gary Sacks, Daisy Coyle, Noah Cooke, David Hammond

**Affiliations:** 1 School of Public Health Sciences, University of Waterloo, Waterloo, ON, Canada; 2 École de Nutrition, Université Laval, Québec City, QC, Canada; 3 MRC Epidemiology, University of Cambridge, Cambridge, England; 4 School of Health & Social Development, Deakin University, Melbourne, VIC, Australia; 5 The George Institute for Global Health, Sydney, NSW, Australia

**Keywords:** Meal-kit, Meal preparation, Dietary patterns, Sustainability

## Abstract

**Objective:**

To assess the frequency and correlates of meal-kit use across five countries using population-level data.

**Design:**

Online surveys conducted in 2022 assessed meal-kit use in the past week. Binary logistic regression models examined sociodemographic and nutrition-related correlates of meal-kit use, including self-reported home meal preparation and cooking skills, commercially prepared meal consumption and healthy eating, weight change and sustainability efforts.

**Setting:**

Canada, Australia, the UK, the USA and Mexico.

**Participants:**

20,401 adults aged 18–100 years.

**Results:**

Overall, 14 % of participants reported using meal-kits in the past week. Use was highest in the USA (18 %) and lowest in Canada (9 %). Meal-kit use was greater among individuals who were younger, male, of minority ethnicity, had high educational attainment, had higher income adequacy or had children living in the household (*P* < 0·01 for all). Use was greater for those who participated in any food shopping (*v*. none), those who prepared food sometimes (3–4 d/week or less *v*. never) and those who reported ‘fair’ or better cooking skills (*v*. poor; *P* < 0·05 for all). Consuming any ‘ready-to-eat’ food (*v*. none) and visiting restaurants more recently (*v*. > 6 months ago; *P* < 0·001 for all) were associated with greater meal-kit use. Eating fruits/vegetables more than 2 times/d and engaging in diet modification efforts were also associated with increased meal-kit use, as was engaging in weight change or sustainability efforts (*P* < 0·001 for all).

**Conclusions:**

Meal-kits tend to be used by individuals who make efforts to support their health and sustainability, potentially valuing ‘convenient’ alternatives to traditional home meal preparation; however, use is concentrated amongst those with higher income adequacy.

Dietary risk factors are among the leading causes of non-communicable diseases, including cardiovascular disease and diabetes^(1)^. Dietary patterns that are low in fruits, vegetables and whole grains and high in salt, unhealthy fats and refined carbohydrates are major contributors to morbidity and mortality^(1)^. Greater home meal preparation frequency is associated with better diet quality among both lower-income and higher-income adults, as indicated by higher Healthy Eating Index scores^(2,3)^, as well as greater adherence to recommended dietary patterns (e.g. Mediterranean and Dietary Approaches to Stop Hypertension) and greater fruit and vegetable intake^(4)^. Higher intake of food prepared in the home is also associated with markers of cardio-metabolic health, including lower adipose mass and waist circumference^(4,5)^. In addition to improved health and diet quality, home meal preparation is also associated with family relationship development and the establishment of stronger cultural identities^(6,7)^. However, home meal preparation has become increasingly less frequent over time, while the consumption of meals commercially prepared outside the home has increased^(8,9)^. The reduced consumption of meals prepared at home may reflect many factors, including general declines in cooking skills and confidence, increased marketing of convenience foods, and lower priority due to competing demands on time^(10–13)^.

Meal-kits have emerged as a hybrid between commercially prepared meals and home meal preparation. Meal-kits are predominantly subscription-based services that provide pre-portioned ingredients for a variety of recipes and are usually delivered directly to consumers’ homes^(14,15)^; however, in some countries such as the USA and the UK, variations of meal-kits are now also available for purchase at major grocery retail stores. Step-by-step recipe instructions are commonly included with the meal-kits, facilitating and expediting home meal preparation^(15,16)^. Subscription-based meal-kits are typically delivered on a weekly basis, permitting fresh ingredients to be included^(17,18)^. Meal-kit companies may additionally have offerings tailored for specific health conditions (e.g. low sodium, low sugar, gluten-free), dietary preferences (e.g. vegan, vegetarian), options for local or organic products, complexity levels, preparation times or cuisines^(19)^.

The meal-kit industry has been rapidly expanding, with a market size valued at over $20 billion in 2022 in the USA^(20)^. This growth was accelerated by the onset of the COVID-19 pandemic, which saw pronounced increases in meal-kit purchasing and home meal preparation^(19,21,22)^. This can, in part, be attributed to closures and capacity limits at restaurants, supply chain shortages reflected in reduced grocery availability, and shifts to working from home^(17,18,23)^. These factors may have increased the time and incentive individuals had to prepare meals at home^(17,24)^. A recent systematic review described growth in the online food sector following the onset of the pandemic chiefly attributed to contactless delivery modes, which became increasingly valued as consumers looked to reduce the number of visits to grocery stores^(18,19,21)^. These changes in food acquisition were reflected in online grocery deliveries and meal delivery applications^(21,23)^.

Meal-kits may offer several unique benefits compared with grocery store purchases^(18)^. Meal-kits may help avoid food waste as consumers can typically indicate the number of portions per meal they wish to be delivered^(14,18)^. A Monte Carlo analysis by researchers out of the USA also showed that meal-kits may have lower greenhouse gas emissions than meals made from food purchased at grocery stores, in large part due to reduced food waste, which offsets emissions attributed to additional packaging^(14)^. Many meal-kit companies also provide recyclable packaging, though emphasis on sustainable practices varies^(25)^. Further, meal-kits have been positioned as a way to potentially increase home meal preparation frequency by combating a lack of time or knowledge, which can impede meal planning^(8,15,26)^. Meal-kits can help reduce the cognitive load of dietary decision making, increasing family members’ participation in home meal preparation and providing an opportunity for building food literacy, cooking skills and confidence^(17,26,27)^. Meal-kits may also provide an opportunity to engage children in home meal preparation by making it easier for children to follow along, enhancing their skills and confidence in the kitchen and facilitating inter-generational socialisation^(17,26,28)^. However, meal-kits may be unaffordable for some consumers, given that their prices are often greater than purchasing equivalent items at grocery stores^(29)^. The cost of purchasing meals-kits is variable based on region, company and food offerings. As of 2025, the cost per serving for meal-kits from leading companies in the USA was on average $10–$12 but ranged as low as $6 to as high as $22 per serving^(30)^.

Despite the growing popularity of meal-kits and their potential to help address the trend in decreasing home meal preparation, there is little research characterising their use at a population level. Most work to date has examined the nutritional composition of meal-kits^(8,31)^ or their acceptability and feasibility for improving food access^(32,33)^ and diet quality^(34,35)^. Few studies have explored the frequency of meal-kit use and the correlates of use. One USA-only study used population-level data to examine meal-kit use during the early months of the COVID-19 pandemic and found that most new users were under 55 years of age, had children in the household and reported eating both more fruits/vegetables and more red/processed meat^(18)^. A recent cross-country study also found that meal-kit users were more likely to be younger, male and have children present in the home^(36)^. While some dichotomous measures of home meal preparation were included, the study largely examined sociodemographic correlates and did not include measures addressing participants’ healthy eating, weight change or sustainability efforts. As such, important questions remain about how meal-kit use relates to broader health and dietary behaviours, as well as the potential role of meal-kits in supporting healthier and more sustainable eating patterns. The present study contributes new evidence by investigating not only the prevalence and correlates of meal-kit use across five countries using self-reported population-level survey data but also associations with dietary behaviours. We examined three primary research questions: (1) How prevalent is meal-kit use, and are there differences in meal-kit use prevalence between countries? (2) What is the sociodemographic profile of meal-kit users, and does it differ by country? and (3) What nutrition-related behaviours are associated with meal-kit use, including home meal preparation and cooking skills, commercially prepared meal consumption, healthy eating efforts, weight change efforts and sustainability efforts?

## Methods

The study used data from the 2022 International Food Policy Study (IFPS) adult survey. The IFPS is a cross-sectional survey conducted in five countries: Canada, Australia, the UK, the USA and Mexico. Data were collected using self-completed web-based surveys administered to adults aged 18–100 years. Respondents were recruited through Nielsen Consumer Insights Global Panel and their partners’ panels. The panels were recruited using both probability and non-probability sampling methods. Email and panellist dashboard application invitations with unique survey access links were shared with a random sample of panellists within each country after targeting for demographics. Potential respondents were screened for eligibility (18–100 years, residence in the target country) and quota requirements based on age and sex. Surveys were conducted in English in Australia and the UK, Spanish in Mexico, English or French in Canada and English or Spanish in the USA. English survey measures were translated into French and Spanish by professional translation companies and subsequently reviewed by bilingual content-experts who were native French and Spanish speakers to confirm nutrition-related terminology. Respondents provided consent prior to survey completion. Respondents received remuneration in accordance with their panel’s usual incentive structure (e.g. points-based or monetary rewards, chances to win prizes). The study was reviewed by and received ethics clearance through the University of Waterloo Research Ethics Board (REB# 30829). Further methodological details are available in the 2022 IFPS Technical Report^(37)^, as well as in the analysis pre-registration published in the Open Science Framework^(38)^.

### Measures

#### Meal-kit use

Respondents were asked a series of questions about food prepared at home in the past 7 d. Regarding meal-kits, respondents were asked, ‘Was any of the food purchased in the past 7 d from a meal-kit delivery service (e.g. HelloFresh, Goodfood)?’ (Yes/No). Different examples of popular meal-kit delivery services were provided in each country (Canada: HelloFresh, Goodfood; Australia: HelloFresh, Marley Spoon; Mexico: Freshybox; UK: HelloFresh, Gousto; USA: HelloFresh, Blue Apron).

#### Home meal preparation and cooking skills

Respondents were asked, ‘How much of the food shopping do you do in your household?’ (Most/Share equally with other(s)/Some, but less than other(s)/None). Respondents were also asked, ‘How often do you prepare a main meal for yourself or others?’ (Never/Only for special occasions/Less than once a week/One or 2 d a week/Some days (3–4 a week)/Most days (5–6 a week)/Every day), and ‘Overall, how would you rate your cooking skills?’ (Poor/Fair/Good/Very Good/Excellent).

#### Commercially prepared meal consumption

Respondents were asked, ‘When was the last time you visited a restaurant (including a fast-food outlet or coffee shop)?’ (Within the last 24 h/Within the last 7 d/ Within the last 3 months/Within the last 6 months/Longer than 6 months ago). Respondents were also asked, ‘Thinking about the meals prepared at home in the past 7 d, what percentage was ‘ready-to-eat’ or ‘box food’ (e.g. microwave, frozen or packaged meals)?’ (numeric: 0–100 %). Responses were grouped into quintiles (0 %/1–25 %/26–50 %/51–75 %/> 75 %).

#### Healthy eating efforts

Respondents were asked a series of questions to assess their total daily fruit and vegetable intake (including fruit, 100 % fruit juice, green leafy or lettuce salad, non-fried potatoes and other vegetables, but excluding fried potatoes and non-100 % fruit juice beverages). Total daily fruit and vegetable intake was tallied and recoded as ‘Less than 1 time per day’, ‘Between 1 and 2 times per day’ or ‘More than 2 times per day’. Respondents were also asked a series of questions to assess if they have made a conscious effort to consume more vegetables and fruits/protein/whole grains or consume less sugary food/processed foods/salt/red or processed meat (e.g. beef, pork, deli meat). A diet modification efforts index was created by summing affirmative responses (range: 0–7).

#### Weight change efforts

Using a ‘select all that apply’ format, respondents were asked, ‘During the past 12 months, have you tried to…’ (Lose weight/Gain weight/Stay the same weight/I have not tried to do anything about my weight).

#### Sustainability efforts

Respondents were asked, ‘Do you make a special effort to eat plant-based protein foods, like beans, lentils, nuts, seeds or soy products like tofu?’ (Yes/No/ I’m not sure what plant-based protein foods are). Respondents were also asked a series of questions about sustainability efforts including if they ‘made a special effort to purchase foods with … low greenhouse gas emissions/organic foods/local foods (food grown in your area)/foods that are in season/fairtrade foods (e.g. fair wages and working conditions)/foods from ethically raised animals/food with less impact on biodiversity (wildlife, habitat loss, soil health)/foods that have less packaging’. A sustainability effort index was created by summing affirmative responses (range: 0–8).

#### Sociodemographic characteristics

Sociodemographic measures included country of survey, sex at birth (Male/Female), age group (18–29 years old/30–44 years old/45–59 years old/60+ years old), ethnicity (Majority/Minority), highest education level completed (Low/Medium/High), perceived income adequacy (assessed by asking, ‘Thinking about your total monthly income, how difficult or easy is it for you to make ends meet?’ Very difficult/Difficult/Neither easy nor difficult/Easy/Very easy) and presence of dependent children in the household (No children < 18 years in household/Children < 18 years in household). Descriptive frequencies for gender are shown in Table 1; however, gender was not integrated into the models due to small cell sizes not appropriate for modelling. Details regarding country-specific response options for ethnicity and education are available in the 2022 IFPS Technical Report^(37)^.

**Table 1.** Sample sociodemographic characteristics, overall and by country, 2022, *n* 20,401 (weighted estimates)

	Overall (*n* 20,401)		Canada (*n* 4,310)		Australia (*n* 4,086)		UK (*n* 4,078)		USA (*n* 3,836)		Mexico (*n* 4,091)	
	%	*n*	%	*n*	%	*n*	%	*n*	%	*n*	%	*n*
Age (years)												
18–29	20·8	4,235	18·9	812	19·8	807	18·0	732	19·2	735	28·1	1,149
30–44	26·9	5,491	25·5	1,099	27·2	1,113	25·3	1,031	25·6	983	31·0	1,266
45–59	25·0	5,090	23·6	1,017	23·5	958	25·2	1,026	24·6	944	28·0	1,146
60+	27·4	5,585	32·1	1,382	29·6	1,208	31·6	1,290	30·6	1,175	13·0	530
Sex at birth												
Male	49·0	9,987	49·8	2,148	49·2	2,010	48·1	1,962	49·5	1,899	48·1	1,968
Female	51·1	10,414	50·2	2,162	50·8	2,076	51·9	2,116	50·5	1,937	52·0	2,123
Gender												
Man	48·5	9,997	49·2	2,098	48·8	2,013	47·7	1,976	48·8	1,874	47·9	2,036
Woman	50·8	10,276	49·5	2,173	50·5	2,049	51·7	2,079	50·4	1,936	51·8	2,039
Trans male/trans man	0·2	25	0·4	7	0·2	5	0·1	3	0·3	8	0·04	2
Trans female/trans woman	0·1	22	0·2	6	0·1	2	0·2	7	0·2	4	0·1	3
Gender diverse	0·4	73	0·8	23	0·5	17	0·3	12	0·4	13	0·2	8
Not stated	0·04	8	0·1	3	0·0	0	0·01	1	0·02	1	0·1	3
Ethnicity^*^												
Majority	75·0	15,292	75·8	3,266	70·3	2,873	85·4	3,481	63·0	2,418	79·5	3,253
Minority	24·2	4,941	23·1	995	29·5	1,207	14·2	580	36·2	1,389	18·8	770
Not stated	0·8	168	1·2	450	0·2	6	0·4	16	0·7	2,850	0·2	68
Income adequacy												
Very difficult	9·9	2,011	9·9	428	8·0	328	7·5	305	11·4	435	12·6	515
Difficult	23·5	4,797	20·3	876	20·2	824	23·1	943	21·9	838	32·2	1,316
Neither easy nor difficult	36·2	7,393	36·4	1,570	35·7	1,460	37·9	1,544	32·2	1,233	38·8	1,586
Easy	20·3	4,144	21·5	927	26·5	1,083	21·4	874	19·5	749	12·5	511
Very easy	9·5	1,927	10·8	464	9·0	369	9·5	389	14·6	559	3·6	145
Not stated	0·6	128	1·1	45	0·5	22	0·5	23	0·6	21	0·4	16
Education^†^												
Low	38·1	7,778	39·8	1,716	37·0	1,511	39·4	1,608	54·3	2,083	21·0	861
Medium	22·8	4,656	32·0	1,381	32·4	1,324	25·3	1,030	10·0	385	13·1	537
High	38·7	7,901	27·8	1,199	30·4	1,241	34·8	1,418	35·3	1,354	65·7	2,688
Not stated	0·3	65	0·3	15	0·2	10	0·5	22	0·4	13	0·1	6
Presence of children in household												
No children < 18 years in household	68·0	13,879	78·7	3,392	69·3	2,833	70·2	2,862	70·9	2,721	50·7	2,072
Children < 18 years in household	32·0	6,522	21·3	918	30·7	1,253	29·8	1,216	29·1	1,115	49·4	2,019

* Ethnicity categories refer to the following: in Canada, ‘majority’ if ‘White (European descent)’ is only category selected, ‘minority’ if any other category selected; in Australia, ‘majority’ if only speak English at home, ‘minority’ if speak a language other than English at home or indicated they are aboriginal or Torres Straight Islander; in the UK and the USA, ‘majority’ if only ‘White’ category is selected, ‘minority’ if selected any other category; and in Mexico, ‘majority’ if do not consider self Indigenous, ‘minority’ if consider self Indigenous.

† Education categories refer to the following: in Canada, ‘low’ if ‘High school diploma/equivalent’ or below, ‘high’ if ‘Bachelor’s degree’ or above; in Australia, ‘low’ if ‘Year 12/ equivalent’ or below, ‘high’ if ‘Bachelor’s degree’ or above; in the UK, ‘low’ if ‘5 + O levels (passes)/CSEs (grade 1)/GCSEs (grades A*-C/9-4), School Certificate, 1 A level/2–3 AS levels/VCEs, Higher Diploma’ or below, ‘high’ if ‘Degree/Higher Degree/ NVQ Level 4–5, HNC, HND, RSA Higher Diploma, BTEC Higher Level/ Professional qualifications’; in the USA, ‘low’ if ‘12th Grade/high school diploma’ or below, ‘high’ if ‘Bachelor’s degree’ or above; and in Mexico, ‘low’ if ‘Normal básica’ or below, ‘high’ if ‘Normal de licenciatura’ or above.

### Data analysis

A total of 22,304 adults completed the survey. Respondents were excluded for the following data quality reasons: ineligible region; invalid response to a data quality question; below minimum survey completion time based on median survey time; and/or invalid responses to at least three of twenty open-ended measures (*n* 1,320). Further, respondents could select ‘Don’t know’ or ‘Refuse to answer’ for all questions except sex at birth and age. ‘Don’t know’ was recoded to ‘No’ for meal-kit use (*n* 174) to preserve sample size and based on the assumption that uncertainty about recent meal-kit use indicates it likely did not occur. A total of 583 respondents with missing data on key variables were excluded, including ‘Don’t know’, ‘Refuse to answer’ and/or system missing where no feasible interpretations could be made. Due to large proportions of missing responses for fruit and vegetable consumption (16·7 %) and ‘ready-to-eat’ food consumption (14·6 %) (including blank values, ‘Don’t know’, ‘Refuse to answer’ and implausible values deleted during data cleaning), a separate missing data category was retained for both variables. Similarly, ‘Don’t know’ and ‘Refuse to answer’ were retained in a separate ‘Not stated’ category for income adequacy (*n* 128), ethnicity (*n* 168) and education (*n* 65). The final analytic sample included 20,401 respondents (Australia: *n* 4,086; Canada: *n* 4,310; Mexico: *n* 4,091; UK: *n* 4,078; USA: *n* 3,836).

Two changes were made to the pre-registered analysis plan^(38)^: (1) total daily fruit and vegetable intake and frequency of ‘ready-to-eat’ meal consumption were analysed as ordinal rather than continuous data due to the large proportions of missing responses noted above, and (2) plant-based consumption was disaggregated from the sustainability efforts index as plant-based consumption may occur for dietary reasons outside of sustainability. No further changes were made to the pre-registered analytic plans published on the Open Science Framework.

Data were weighted with post-stratification sample weights constructed using a raking algorithm with population estimates from the census in each country based on age group, sex, region, ethnicity (except in Canada) and education (except in Mexico). Weights were rescaled to the unweighted sample size in each country; estimates reported are weighted unless otherwise specified. Descriptive statistics were used to characterise variables. Cross-sectional analysis was conducted using binary logistic regression; two-way interactions were tested between country and sociodemographic variables, with 95 % CI and adjusted odds ratios (AOR) reported. All models were adjusted for country, age, sex at birth, ethnicity, education, income adequacy and presence of dependent children in the household. All statistical analyses were conducted using SAS Studio statistical software, version 9.4.

## Results

### Sociodemographic characteristics

The sociodemographic characteristics of the sample are shown in Table 1, overall and by country.

### Differences in meal-kit use by country and sociodemographic characteristics

As shown in Table 2, 13·7 % of respondents reported using meal-kits in the past 7 d. Meal-kit use was lowest in Canada (8·8 %), followed by the UK (11·6 %), Australia (14·8 %) and Mexico (15·7 %), with use highest in the USA (18·0 %). Use in the UK, Australia and Mexico was not significantly different from each other in unadjusted models; however, after adjusting for covariates, respondents in Australia had greater odds of meal-kit use than those in Mexico (AOR: 1·21, CI: 1·02, 1·42, *P* = 0·030).

**Table 2.** Meal-kit use in past 7 d by sociodemographic factors, 2022, *n* 20,401 (weighted estimates)

	% using meal-kit^*^	*n*	AOR^†^	95 % CI^‡^	*P* value
Overall	13·7	2,784	–	–	–
Country					
Canada	8·8	377	Ref	–	–
Australia	14·8	604	1·50	1·25, 1·79	< 0·001
UK	11·6	472	1·32	1·10, 1·59	0·003
USA	18·0	689	2·02	1·68, 2·42	< 0·001
Mexico	15·7	642	1·24	1·03, 1·50	0·024
Age (years)					
18–29	22·7	960	Ref	–	–
30–44	23·6	1,293	0·68	0·60, 0·78	< 0·001
45–59	8·0	408	0·27	0·23, 0·32	< 0·001
60+	2·2	123	0·10	0·07, 0·13	< 0·001
Sex at birth					
Female	9·6	996	Ref	–	–
Male	17·9	1,788	2·03	1·83, 2·26	< 0·001
Ethnicity					
Majority	11·0	1,676	Ref	–	–
Minority	21·9	1,080	1·54	1·37, 1·74	< 0·001
Not stated	17·3	28	1·37	0·78, 2·42	0·272
Income adequacy					
Very difficult	10·4	210	Ref	–	–
Difficult	10·1	482	0·84	0·67, 1·05	0·133
Neither easy nor difficult	11·5	851	0·98	0·79, 1·21	0·816
Easy	17·7	733	1·53	1·23, 1·90	< 0·001
Very easy	25·6	494	2·52	2·00, 3·17	< 0·001
Not stated	10·7	14	0·86	0·38, 1·94	0·715
Education					
Low	9·7	752	Ref	–	–
Medium	9·8	454	1·08	0·91, 1·27	0·384
High	19·9	1,569	1·68	1·47, 1·92	< 0·001
Not stated	13·4	9	1·38	0·61, 3·12	0·446
Presence of children in household					
No children < 18 years in household	7·5	1,040	Ref	–	–
Children < 18 years in household	26·8	1,744	2·88	2·56, 3·22	< 0·001

* Percentage of respondents who reported using a meal-kit in past 7 d.

† Adjusted OR from a logistic regression model adjusted for country, age, sex at birth, ethnicity, education, income adequacy and presence of dependent children in the household.

‡ 95 % CI.

Among all countries pooled, past 7 d meal-kit use varied by age and other sociodemographic characteristics. In models adjusting for covariates, there was a stepwise decrease in odds of using meal-kits across the age groups, with respondents aged 18–29 (22·7 %) having the greatest odds of meal-kit use (*P* < 0·001 for all). Males (17·9 %) reported greater odds of using meal-kits compared with females (9·6 %, AOR: 2·03, CI: 1·83, 2·26, *P* < 0·001). Compared with those who were of ‘majority’ ethnicity (11·0 %), those who were of ‘minority’ ethnicity (21·9 %) had greater odds of using meal-kits (AOR: 1·54, CI: 1·37, 1·74, *P* < 0·001). Odds of meal-kit use were also higher for those reporting ‘easy’ (17·7 %, AOR 1·53, CI: 1·23, 1·90) and ‘very easy’ (25·6 %, AOR: 2·52, CI: 2·00, 3·17) income adequacy compared with ‘very difficult’ (10·4 %, *P* < 0·001 for both). No differences in meal-kit use were observed between those at the middle and lower ends of income adequacy. Those who reported ‘high’ education (19·9 %) had greater odds of meal-kit use compared with those reporting ‘low’ education (9·7 %, AOR: 1·68, CI: 1·47, 1·92, *P* < 0·001), with no differences between the ‘medium’ and ‘low’ education levels. Finally, odds of meal-kit use were greater for those who had children under 18 years in the household (26·8 %), compared with those who did not have children present (7·5 %, AOR 2·88, CI: 2·56, 3·22, *P* < 0·001).

**Fig. 1:**
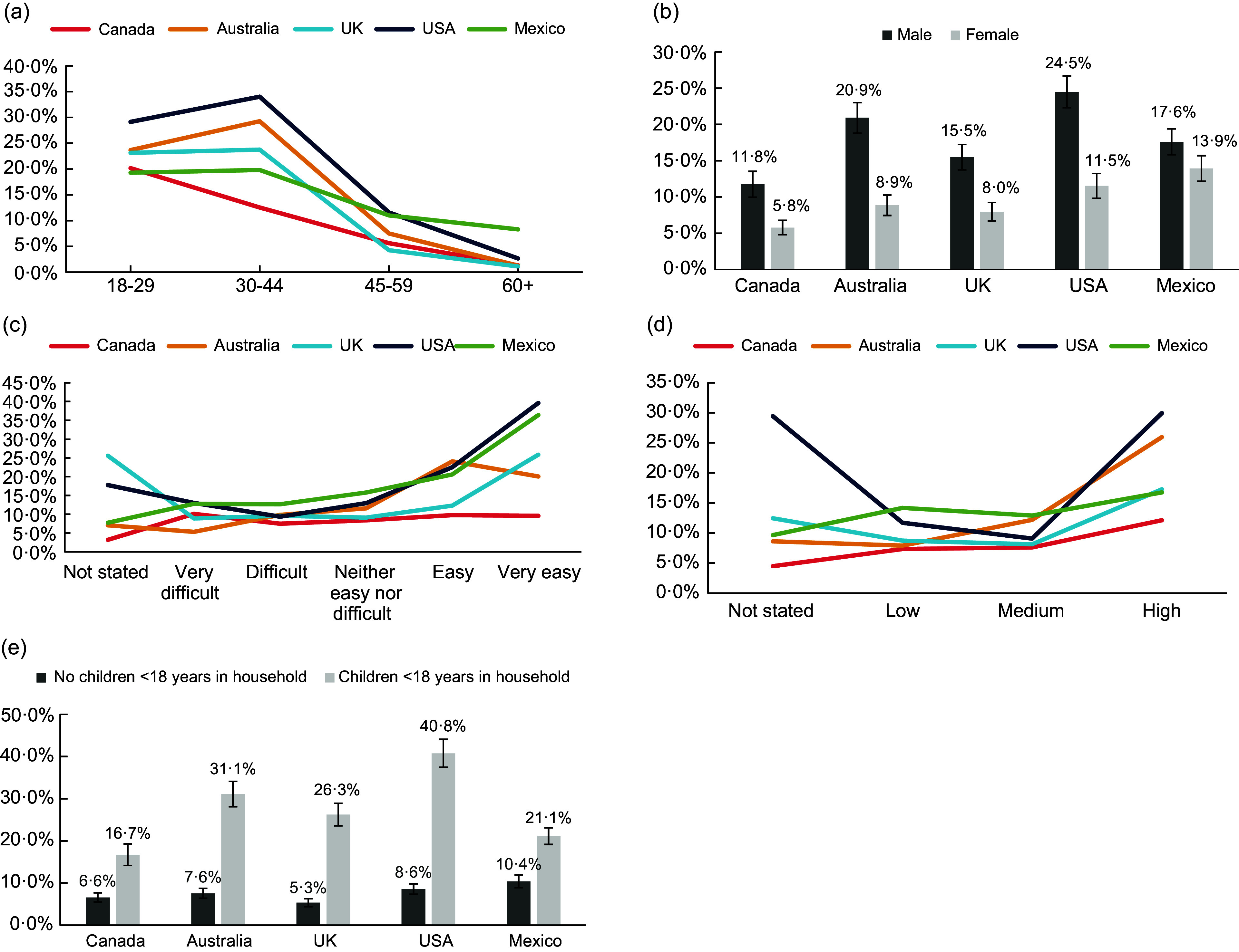
Interactions of meal-kit use in past 7 d with sociodemographic characteristics, by country, *n* 20,401 (weighted estimates). (a) Interaction of meal-kit use with age, by country. (b) Interaction of meal-kit use with sex at birth, by country. (c) Interaction of meal-kit use with income adequacy, by country. (d) Interaction of meal-kit use with education, by country. (e) Interaction of meal-kit use with presence of children in household, by country.

In terms of interactions, differences in sociodemographic trends between countries were observed for age (F = 6·17, *P* < 0·001), sex (F = 6·35, *P* < 0·001), income adequacy (F = 3·07, *P* < 0·001), education (F = 3·35, *P* < 0·001) and presence of children in household (F = 5·92, *P* < 0·001). As Fig. 1(a) shows, while those aged 18–29 in Canada had greater meal-kit use than those aged 30–44, the opposite was observed in Australia and the USA, and no differences were observed in the UK and Mexico. Across all countries, males had greater odds of using meal-kits than females; however, the magnitude of difference was smallest in Mexico and greatest in the USA and Australia (Fig. 1b). As Fig. 1(c) demonstrates, the difference in meal-kit use between lower and higher income adequacy was most pronounced in the USA and Mexico. In Canada, meal-kit use was low across all income adequacy categories and was largely not significantly different by category. Additionally, in Australia, meal-kit use appeared to peak in the ‘easy’ income adequacy category, as compared with the peak at ‘very easy’ for other countries. Across all countries, meal-kit use was highest for those with ‘high’ education, as shown in Fig. 1(d). The magnitude of difference between the ‘low’ and ‘high’ education levels was most pronounced in the USA and Australia and least pronounced in Mexico. Finally, the trend of those with children present in the household having greater odds of using meal-kits was observed across all countries (Fig. 1e). However, the magnitude of difference was greatest in the USA, and lowest in Canada and Mexico. The interaction of country with ethnicity was not statistically significant (F = 1·76, *P* = 0·080).

### Association between meal-kit use and home meal preparation and cooking skills

As shown in Table 3, those who did any shopping had greater odds of using meal-kits than those who did ‘none’ of the shopping (*P* < 0·01 for all). Those who reported preparing food ‘some days (3–4 per week)’ or less (up to only preparing food for ‘special occasions’) had greater odds of using meal-kits than those who ‘never’ prepared food (*P* < 0·01 for all). Differences in the frequency of meal-kit use between those who prepared food ‘most days (5–6 per week)’ (9·6 %, *P* = 0·439) or ‘every day’ (6·4 %, *P* = 0·580) with those who ‘never’ prepared food (4·9 %) were not statistically significant. Finally, those who rated their cooking skills as ‘fair’ or better had greater odds of using meal-kits compared with those who rated their skills as ‘poor’ (*P* < 0·05 for all).

**Table 3.** Meal-kit use in past 7 d by home meal preparation, cooking skills and commercially prepared meal consumption, 2022, *n* 20,401 (weighted estimates)

	% Using Meal-Kit^*^	*n*	AOR^†^	95 % CI^‡^	*P* value
Food shopping					
None	4·6	17	Ref	–	–
Some, but less than other(s)	13·3	171	3·00	1·38, 6·54	0·006
Share equally with other(s)	16·5	806	4·44	2·08, 9·46	< 0·001
Most	12·9	1,790	4·34	2·04, 9·25	< 0·001
Food preparation					
Never	4·9	34	Ref	–	–
Only for special occasions	24·8	354	4·56	2·78, 7·47	< 0·001
Less than once per week	25·2	387	4·27	2·62, 6·98	< 0·001
One or 2 d per week	20·7	576	2·90	1·79, 4·68	< 0·001
Some days (3–4 per week)	16·3	608	1·88	1·16, 3·03	0·010
Most days (5–6 per week)	9·6	500	1·21	0·75, 1·96	0·439
Every day	6·4	325	0·87	0·53, 1·42	0·580
Cooking skills					
Poor	5·7	57	Ref	–	–
Fair	10·0	456	1·51	1·05, 2·17	0·028
Good	13·0	942	2·18	1·53, 3·12	< 0·001
Very good	14·9	789	2·75	1·10, 3·97	< 0·001
Excellent	23·5	540	4·72	3·22, 6·90	< 0·001
Last restaurant visit					
Longer than 6 months ago	3·8	72	Ref	–	–
Within the last 6 months	17·3	143	3·64	2·32, 5·71	< 0·001
Within the last 3 months	14·7	920	2·76	1·90, 3·99	< 0·001
Within the last 7 d	13·7	1,182	2·14	1·48, 3·09	< 0·001
Within the last 24 h	16·8	467	2·52	1·73, 3·66	< 0·001
Processed ‘ready-made’ meal intake					
0 %	1·5	77	Ref	–	–
1–25 %	9·6	639	5·03	3·83, 6·61	< 0·001
26–50 %	23·1	917	11·43	8·72, 14·99	< 0·001
51–75 %	35·2	432	20·93	15·52, 28·21	< 0·001
75+ %	23·5	541	14·17	10·61, 18·91	< 0·001
Missing/Don’t know/Not reported	14·6	178	8·56	6·05, 12·12	< 0·001

* Percent of respondents who reported using a meal-kit in past 7 d.

† Adjusted OR from a logistic regression model adjusted for country, age, sex at birth, ethnicity, education, income adequacy and presence of dependent children in the household.

‡ 95 % CI.

### Association between meal-kit use and commercially prepared meal consumption

As also shown in Table 3, those who last visited a restaurant within the past 6 months or more recently had greater odds of using meal-kits compared with those whose last visit was longer than 6 months ago (*P* < 0·001 for all). There was a trend of increased meal-kit use across the middle three restaurant visit frequency categories (last 7 d to last 3 months (13·7 % *v*. 14·7 %, *P* < 0·001); last 3 months to last 6 months (14·7 % *v*. 17·3 %, *P* = 0·054)), wherein use increased with decreasing recency of visit; however, use among those who visited a restaurant within the last 24 h was similar to those who visited in the last 3 months (*P* = 0·241). In terms of processed ‘ready-to-eat’ meal intake in the past 7 d, those who consumed at least 1 % of intake from ‘ready-to-eat’ meals had greater odds of meal-kit use compared with those who reported not consuming ‘ready-to-eat’ meals (*P* < 0·001 for all). Those who reported 51–75 % intake from ‘ready-to-eat’ meals (35·2 %) had the greatest odds of using meal-kits, compared with those who reported not consuming ‘ready-to-eat’ meals (AOR: 20·93, CI: 15·52, 28·21, *P* < 0·001).

### Association between meal-kit use and healthy eating efforts

As shown in Table 4, respondents who ate fruits/vegetables ‘more than two times per day’ (14·3 %) had greater odds of using meal-kits compared with those who ate fruits/vegetables ‘less than 1 time per day’ (6·9 %, AOR: 1·95, CI: 1·45, 2·61, *P* < 0·001). No differences were observed between those who reported eating fruits/vegetables ‘between 1 and 2 times per day’ (8·3 %) and ‘less than 1 time per day’ (*P* = 0·388). Further, respondents who used meal-kits engaged in an average of 3·97 diet modification efforts (sd: 2·13), compared with an average of 3·80 for those who did not use meal-kits (sd: 2·29, range: 0–7). Respondents who engaged in more diet modification efforts had greater odds of using meal-kits (AOR: 1·06, CI: 1·03, 1·08, *P* < 0·001).

**Table 4.** Meal-Kit use in past 7 d by healthy eating, weight change and sustainability efforts, 2022, *n* 20,401 (weighted estimates)

	% using meal-kit^*^	*n*	AOR^†^	95 % CI^‡^	*P* value
Healthy eating efforts					
Fruit and vegetable consumption					
Less than 1 time per day	6·9	70	Ref	–	–
Between 1 and 2 times per day	8·3	219	1·16	0·83, 1·61	0·388
More than 2 times per day	14·3	1,757	1·95	1·45, 2·61	< 0·001
Missing/Don’t know/Not reported/Deleted (total fruit/vegetable intake >= 100)	16·7	738	2·16	1·60, 2·92	< 0·001
Diet modification efforts index					
Mean	3·97		1·06	1·03, 1·08	< 0·001
sd	2·13				
Weight change efforts					
Did not try to do anything about weight	5·4	287	Ref	–	–
Tried to lose weight	11·4	983	2·06	1·74, 2·45	< 0·001
Tried to gain weight	30·8	571	4·30	3·52, 5·26	< 0·001
Tried to stay the same weight	20·0	892	2·88	2·42, 3·43	< 0·001
Tried to both lose and gain weight	37·4	51	4·24	2·50, 7·22	< 0·001
Sustainability efforts					
Plant-based consumption					
No	7·7	960	Ref	–	–
I’m not sure what plant-based foods are	16·6	64	1·99	1·36, 2·92	< 0·001
Yes	23·5	1,760	2·48	2·21, 2·79	< 0·001
Sustainability efforts index					
Mean	4·64		1·11	1·09, 1·14	< 0·001
sd	2·61				

* Percent of respondents who reported using a meal-kit in past 7 d.

† Adjusted OR from a logistic regression model adjusted for country, age, sex at birth, ethnicity, education, income adequacy and presence of dependent children in the household.

‡ 95 % CI.

### Association between meal-kit use and weight change efforts

As also shown in Table 4, respondents who reported engaging in any weight change efforts had greater odds of meal-kit use, compared with those who ‘did not try to do anything about their weight’ (*P* < 0·001 for all).

### Association between meal-kit use and sustainability efforts

As additionally shown in Table 4, respondents who reported making an effort to consume plant-based food (23·5 %) or were ‘not sure what plant-based foods are’ (16·6 %) had greater odds of using meal-kits than those who did not report making an effort to consume plant-based foods (7·7 %, *P* < 0·001 for both). Further, respondents who reported using meal-kits engaged in an average of 4·64 sustainability efforts (sd: 2·61), compared with an average of 3·44 for those who did not use meal-kits (sd: 2·70, range: 0–8). Respondents who engaged in more sustainability efforts had greater odds of using meal-kits (AOR: 1·11, CI: 1·09, 1·14, *P* < 0·001).

## Discussion

To our knowledge, this is the first study to examine the prevalence of meal-kit use among countries and in relation to individual correlates of use. Across countries, approximately 14 % of respondents reported using meal-kits in the past 7 d in 2022, with use lowest in Canada (9 %) and highest in the USA (18 %). This aligns with trends reported in USA consumer surveys, wherein 9 % of respondents reported purchasing a meal-kit in 2018 and 25 % indicated they would be interested in purchasing one in the future^(14)^. Research out of the UK suggests that up to 30 % of consumers used meal-kits during the early phase of the COVID-19 pandemic, though more recent estimates are unavailable^(39)^. The difference in use between countries is surprising given the similarities in food environments and comparable costs per portion (excluding gourmet/specialty meal-kits)^(40,41)^. Possible explanations may be related to the variety of meal-kit offerings, delivery reach, and relative cost compared with groceries; further research is needed to examine these factors.

### Cost and income adequacy

Across all countries, except Canada, where meal-kit use remained low for all income adequacy categories, pronounced differences in meal-kit use were observed between lower and higher income adequacies. Meal-kit use was associated with higher income adequacy and greater educational attainment. This is in line with research from the USA showing the majority of meal-kit users have incomes above USD $100,000 and college degrees^(18)^. Although meal-kits may be more affordable than eating at a restaurant or ordering take-away, they can be more expensive than purchasing the equivalent ingredients in a grocery store and may yield fewer servings due to pre-portioning^(29)^. Meal-kits often include some fresh fruits/vegetables, meaning they may be more expensive than simpler meals or meals using canned, frozen or processed products and were shown to be perceived as expensive in an Australian qualitative study^(8,17,29)^. These factors may explain why use appears to be more prevalent amongst those with higher income adequacy.

Meal-kit use was also greater among individuals who were of minority ethnicity. Neighbourhoods with greater amounts of minority ethnicity and lower socioeconomic status residents have been found to have lower density of grocery stores and higher density of convenience stores compared with neighbourhoods with majority ethnicity and higher socioeconomic status residents in the USA and Canada^(42,43)^. A recent study exploring a USA-based community-led meal-kit business found that individuals of minority ethnicity were more likely to live in areas with fewer healthy food options and were open to using meal-kits as a potential way to fill this gap^(32)^. There are several non-governmental organisations in the UK offering meal-kits to low-income consumers at lower prices^(44)^. Though meal-kits may play a role in supporting food availability for minority and lower-income populations^(45,46)^, there is a need to explore the extent to which access to commercial meal-kits is constrained by cost and how they may be leveraged in improving food access.

### Home meal preparation, cooking skills, and convenience

Meal-kit use was more common among younger age groups, in line with previous research with USA populations^(18)^. Meal-kits are commonly sold digitally through websites and phone applications, which may have greater appeal for younger consumers^(16)^. Older adults (60+) may also have fewer competing demands for time (due to retirement), facilitating preparation ‘from scratch’. Further, UK adults aged 19–34 years have been found to report lower confidence and less familiarity with home meal preparation compared with older generations who have historically had less access to commercially prepared meals^(47)^. Younger adults may thus be more inclined to use meal-kits, given that most recipes in meal-kits can be considered ‘easy’ or ‘very easy’ to follow^(8)^. Yet, many meal-kits still require some level of cooking skills and access to relevant equipment^(8)^, as evidenced by the positive association of meal-kit use with cooking skills identified in our study. Younger adults are known to be less likely to have, or have had, children at home, necessitating frequent home meal preparation and subsequently skills development^(47)^, consistent with the drop in meal-kit use observed for respondents over the age of 45. In our study, meal-kit use was found to be greater among individuals with children living in the household, in line with literature showing that families with children are more likely to prepare meals at home^(3,18)^. As meal-kits are often pre-portioned and contain step-by-step recipe instructions (often times with photos), they may reduce the cognitive load of dietary decision making^(17,26)^, which may be particularly important for parents of younger children^(17,26)^.

Although women globally are more likely to be involved in home meal preparation overall^(3,17)^, in our study, men were more likely to report using meal-kits. On average, men tend to work longer paid hours than women^(48,49)^, while women are more likely to engage in unpaid household work^(50,51)^; those who work longer paid hours have been demonstrated to be less likely to prepare meals^(3,17)^. Men generally also tend to have less well-developed food preparation skills than women^(52)^. By facilitating the meal preparation process, meal-kits may have the potential to influence traditional gender norms around food preparation roles^(3,17)^; it may be possible that women are initiating the purchasing of meal-kits as a way of involving male partners^(17)^.

Notably, meal-kit use was greater among respondents who participated in at least some food shopping but prepared meals less often. Meal-kit use was associated with any processed ‘ready-to-eat’ meal intake (e.g. microwave meals, frozen dinners) and visiting restaurants (including fast-food outlets, coffee shops) within the past half year; meal-kit use was lowest for those who last visited a restaurant longer than 6 months ago. This is in contrast to a recent qualitative study by Fraser *et al.*, wherein Australian participants reported meal-kits displacing commercially prepared takeaway and ready-to-eat foods, particularly during busy periods^(17)^. However, participants in the study noted that during periods of lowered mood or heightened stress, meal-kits were not able to address the common barriers of reduced motivation to prepare meals at home^(17)^. In these scenarios, participants relied on takeaway and processed ready-to-eat foods requiring minimal physical/mental effort^(17)^. These findings highlight a key benefit of meal-kits: convenience, which has the potential to increase both meal-kit and commercially prepared meal intake. A recent USA study characterised meal-kit consumers into five profiles based on their values, among which ‘convenience-oriented’ consumers had the greatest intention to purchase meal-kits (and were more likely to be male, married and 35–44 years)^(53)^. As such, meal-kits may be used in combination with commercially prepared meals by those looking for a more ‘convenient’ alternative to traditional home meal preparation. This hybrid approach to home meal preparation may impart ‘easier’ preparation and cooking skills that could be building blocks for further skill development^(27,34)^. This speaks to a potential bidirectional relationship between meal-kit use and home meal preparation, where (1) some meal preparation skills are needed to initiate use and (2) meal-kit use may encourage home meal preparation, potentially developing skills and decreasing out-of-home meal consumption in the long term.

### Health and other considerations of use

Meal-kits may appeal to individuals who are health-conscious but may require some assistance in achieving health goals. In our study, meal-kit use was associated with greater fruit/vegetable consumption and greater likelihood of engaging in diet modification efforts, consistent with other literature^(18)^. A recent Australian study examining meal-kit use found that the majority of respondents believed meal-kits to be aligned with Australia’s national dietary guidelines and to be relatively nutrient dense^(17)^. This is consistent with research showing that meal-kits generally provide appropriate servings of macro- and micro-nutrients, including servings of fruits and vegetables equivalent to/exceeding average daily consumption for Australian adults in one meal^(54,55)^. Owing to the wide range of recipe options available from most meal-kit delivery services^(19)^, meal-kits may expand users’ agency to prepare and enjoy healthy meals and facilitate transitioning to a healthier diet. As such, it is possible that (1) meal-kits support healthier diets by providing added sources of fruits and vegetables (as demonstrated in previous studies^(34)^), (2) individuals with healthier diets may seek out meal-kits as part of their diets or (3) meal-kits contribute to a virtuous cycle of improved diet outcomes through both. Indeed, respondents in our study who did not try to do anything about their weight were the least likely to use meal-kits, indicating that meal-kits may be part of a conscious effort to change dietary patterns.

Consuming plant-based products and engaging in sustainability efforts were similarly associated with meal-kit use. While meal-kits have been associated with some negative environmental impacts due to additional packaging and home delivery^(14)^, one study out of the USA suggests that meal-kits may have lower life cycle environmental impacts than equivalent items purchased from grocery stores^(14)^. This may be due to lower emissions, owing to supply chain logistics, as well as reduced food waste due to pre-portioning and reduced impulse purchasing in stores^(8,14)^. In both the case of meal-kits and grocery stores, one of the largest contributors to emissions was food production itself^(14)^. On average, meal-kits were estimated to have 33 % lower emissions per meal compared with the same meal purchased from a grocery store^(14)^. A separate USA study produced more mixed findings, highlighting reusable packaging and potential reductions in the number of weekly trips to the grocery store as ways to further reduce the environmental impacts of meal-kits^(56)^. More research is needed to assess the environmental impact of meal-kits in comparison with not just equivalent grocery store meals but also commercially prepared meals.

### Strengths and limitations

This study is subject to limitations common to survey research. First, recruitment was conducted using non-probability-based sampling. As such, the findings are not able to provide nationally representative estimates. Second, meal-kit use was measured as a binary outcome for past 7 d only. This shorter time frame may under-estimate use. Given the relative convenience of meal-kits, individuals may order meal-kits sporadically during particularly busy periods, rather than consistently on a weekly basis. This is particularly likely for low- and middle-income individuals, whereas those with higher income may be able to afford ordering meal-kits weekly. Future research measuring meal-kit use over a more prolonged period may be better able to capture ordering prevalence. Additionally, although the meal-kit measure was not formally validated, it was developed by content-experts and reviewed by investigators in each survey country to ensure relevance. Third, respondents who indicated they did not prepare any food at home were not asked about ‘ready-to-eat’ meal consumption (e.g. frozen meals) and were grouped in with the ‘missing/don’t know/refuse’ category rather than the 0 % category as intake could not be confirmed. This may have potentially underestimated ‘ready-to-eat’ intake as respondents may not have identified ‘ready-to-eat’ meals with home meal preparation. Fourth, nutrition-related behaviours were not assessed in relation to meal-kit use specifically. Due to the cross-sectional nature of the study, the study results are limited to associations, and the direction of causality cannot be interpreted. As such, while we have described associations in light of existing literature, further research is needed to assess differences in nutrition behaviours as a result of meal-kit purchasing directly. Fifth, while this study examined meal-kit use across countries generally, it did not address potential between-country differences in the composition or social meaning of meal-kits. There is thus a need for more international evaluations of meal-kit use and composition to further understand how use may differ between and within countries. Finally, further research is needed to examine changes in meal-kit use over time, given continuing growth in the online food sector.

### Conclusions

Meal-kits are an emerging food source across a range of countries, to varying extents. Meal-kits appear to be most commonly purchased by individuals who have a greater interest in sustainable and healthy dietary patterns. Convenience appears to be an important aspect of meal-kits, as meal-kit use is greater for those who purchase commercially prepared meals and prepare meals less frequently, despite some cooking skills. Future research should examine the extent to which meal-kits may be promoting or displacing home meal preparation more widely, including examining home meal preparation from ‘scratch’. Additionally, though meal-kits present a novel way to make eating at home more accessible, they remain relatively expensive and primarily purchased by those with higher income adequacy. Further research is needed to understand the extent to which cost limits access to meal-kits and how access may be improved.
